# Supplementary material for: Enhancing prehospital decision-making: exploring user needs and design considerations for clinical decision support systems
Source: BMC Med Inform Decis Mak. 2025 Jan 17;25:31. doi: 10.1186/s12911-024-02844-1 (PMC11742207; doi:10.1186/s12911-024-02844-1)
Supplement: Supplementary file 1 — Supplementary Material 1 [file 12911_2024_2844_MOESM1_ESM.docx]

## Interview Protocol for EMS Decision Support

## Introduction

- Thank you for taking time out of your schedule to talk with us today.
- We’re a team of researchers from Pace University. The overall goal of our project is to improve pre-hospital care quality and efficiency through our application designs, and now we are designing a pre-hospital clinical decision support system for that goal.
- In today’s interview, we’d like to ask you a few questions regarding your experience and thoughts of using clinical decision support systems in your work. Please feel free to ask any questions or share any additional ideas along the way.

## Participant Rights

- Please review the consent form and let us know if you have any questions.
- Just to quickly cover your rights as a participant for your protection.
- Your participation in this session is voluntary and you may choose to leave at any time.
- We will be audio recording today’s session for data analysis purposes only.
- Your identity will not be associated with any data or used in any subsequent presentations or publications.

Do we have your permission to start the recording?

## Questions

1. **Typical Decision-Making in Pre-Hospital Care**

- What kinds of decisions do you typically make during pre-hospital care?
  - **Follow-up:** What types of decisions do you consider the most critical in pre-hospital care? Why?
  - **Follow-up:** Which decisions do you personally find the most challenging? Can you explain why?

1. **Current Tools for Decision Support**

- Are you currently using any paper or digital tools to support decision-making during pre-hospital care?
  - **If yes:** Can you describe your experience using these tools? (E.g., ease of use, reliability, etc.)
  - **If no:** Have you seen colleagues using any decision support tools? If so, what has been their experience?

1. **Decision Support Features in EHR Systems**

- Does your current Electronic Health Record (EHR) system include any decision support features?
  - **If yes:** What decision-support features are available? How do they support your work?

1. **Suggestions for Useful Decision-Support Features**

- In your opinion, what decision-making features would be most helpful to EMS providers with varying levels of experience?
  - **If needed, provide examples and ask for feedback on each:**
    - **Diagnosis Assistance:** Automatically suggest possible diagnoses when specific criteria are met.
    - **Medication Information Search:** Look up medication information to understand its use in a patient’s care.
    - **Protocol Lookup:** Allow quick access to medical protocols for specific conditions.
    - **Weight Estimation:** Use computer vision technology to estimate a patient's weight.
    - **Medication Dosage Lookup:** Allow quick check of medication dosage for a specific patient (e.g., pediatric patients).
    - **Treatment Assistance:** Automatically suggest treatment or medication recommendations based on the patient's condition.
    - **Hospital/Destination Recommendations:** Provide suggestions for the nearest or the most appropriate hospital for transporting the patient, based on GPS, hospital capacity, and available services.
    - **Alerts:** Trigger alerts for issues like incorrect medication dosage, critical vital signs, or deviations from standard workflows.
      - **If this is deemed useful:** What form of alerts would you prefer? (e.g., visual alerts like flashing red, acoustic alerts like beeping, haptic alerts like vibrations)
    - **Other Features:** Are there any additional decision support features you think would be beneficial?

1. **General Opinion on Decision Support Tools**

- What is your general opinion on using decision support tools in your work? Do you see them as beneficial or burdensome?

1. **Considerations for Decision Support Tool Design**

- What key considerations should be addressed to create a useful decision support tool that seamlessly integrates into your work? (E.g., ease of use, speed, accuracy, etc.)
